# ^11^C-hydroxy-ephedrine-PET/CT in the Diagnosis of Pheochromocytoma and Paraganglioma

**DOI:** 10.3390/cancers11060847

**Published:** 2019-06-19

**Authors:** Achyut Ram Vyakaranam, Joakim Crona, Olov Norlén, Per Hellman, Anders Sundin

**Affiliations:** 1 Section of Radiology & Molecular Imaging, Department of Surgical Sciences, Uppsala University, Akademiska Sjukhuset, SE-751 85 Uppsala, Sweden; anders.sundin@radiol.uu.se; 2 Department of Medical Sciences, Uppsala University, Akademiska Sjukhuset, SE-751 85 Uppsala, Sweden; joakim.crona@medsci.uu.se; 3 Department of Immunology, Genetics and Pathology, Uppsala University, Akademiska Sjukhuset, SE-751 85 Uppsala, Sweden; 4 Department of Surgical Sciences, Uppsala University, Akademiska Sjukhuset, SE-751 85 Uppsala, Sweden; olov.norlen@surgsci.uu.se (O.N.); per.hellman@surgsci.uu.se (P.H.)

**Keywords:** pheochromocytoma, paraganglioma, PET-CT, ^11^C-hydroxy-ephedrine, adrenal incidentaloma

## Abstract

Pheochromocytomas (PCC) and paragangliomas (PGL) may be difficult to diagnose because of vague and uncharacteristic symptoms and equivocal biochemical and radiological findings. This was a retrospective cohort study in 102 patients undergoing ^11^C-hydroxy-ephedrine (^11^C-HED)-PET/CT because of symptoms and/or biochemistry suspicious for PCC/PGL and/or with radiologically equivocal adrenal incidentalomas. Correlations utilized CT/MRI, clinical, biochemical, surgical, histopathological and follow-up data. ^11^C-HED-PET/CT correctly identified 19 patients with PCC and six with PGL, missed one PCC, attained one false positive result (nodular hyperplasia) and correctly excluded PCC/PGL in 75 patients. Sensitivity, specificity, positive and negative predictive values of ^11^C-HED-PET/CT for PCC/PGL diagnosis was 96%, 99%, 96% and 99%, respectively. In 41 patients who underwent surgical resection and for whom correlation to histopathology was available, the corresponding figures were 96%, 93%, 96% and 93%, respectively. Tumor ^11^C-HED-uptake measurements (standardized uptake value, tumor-to-normal-adrenal ratio) were unrelated to symptoms of catecholamine excess (*p* > 0.05) and to systolic blood pressure (*p* > 0.05). In PCC/PGL patients, norepinephrine and systolic blood pressure increased in parallel (*R^2^* = 0.22, *p* = 0.016). ^11^C-HED-PET/CT was found to be an accurate tool to diagnose and rule out PCC/PGL in complex clinical scenarios and for the characterization of equivocal adrenal incidentalomas. PET measurements of tumor ^11^C-HED uptake were not helpful for tumor characterization.

## 1. Introduction

Pheochromocytoma (PCC) and paraganglioma (PGL) are rare, catecholamine-producing chromaffin cell tumors arising from the adrenal gland or extra-adrenal paraganglia, respectively [1,2,3]. These tumors may be difficult to recognize due to uncharacteristic symptomatology mimicking that of more common disorders. The diagnosis in patients with suspected PCC/PGL can be established by biochemical testing, revealing high levels of plasma/urinary catecholamines and/or catecholamine metabolites [4,5].

However, a vast majority of patients who require imaging work-up and hormonal testing to exclude PCC/PGL have so-called “adrenal incidentalomas,” found on imaging that was performed because of reasons other than adrenal disease. Due to the rapid increase in the use of cross-sectional imaging and the fact that incidentalomas are found in approximately 5% of CT examinations, the characterization and follow-up of adrenal incidentalomas places increasing demands on healthcare resources [6,7]. Tumor attenuation measurements on non-contrast-enhanced CT or in/out-of-phase MRI, will in many patients allow for the characterization of lipid-rich adrenocortical adenomas, and the majority of the remaining uncharacterized incidentalomas can later be discarded based on the absence of tumor growth on follow-up imaging [8,9].

Another challenging scenario is when a primary tumor cannot be localized on CT/MRI in biochemically suspected PCC/PGL. In these situations, CT/MRI may be supplemented by functional imaging to depict and characterize the tumor and increase the imaging sensitivity and specificity [10]. Several nuclear medicine imaging tracers are available for positron emission tomography (PET)[11] and scintigraphy, including single-photon emission tomography (SPECT). The general PET-tracer ^18^F-fluoro-deoxy-glucose (^18^F-FDG) provides information on tumor metabolism [12], whereas more specialized tracers target specific pathways such as ^123^I-meta-iodo-benzoguanidine (^123^I-MIBG) [13] for scintigraphy,^18^F-dihydroxy-fluoro-L-phenylalanine (^18^F-DOPA) [14,15,16,17,18,19],^18^F-Dopamine (^18^F-DA) [20,21,22,23],^68^Ga-DOTA-somatostatin analogs [15,24,25,26,27] and ^11^C- hydroxy ephedrine (^11^C-HED) [28,29,30,31,32] for PET/CT. ^11^C-HED is a norepinephrine analog that binds to the norepinephrine transporter and has previously shown high diagnostic sensitivity and specificity in patients with PCC/PGL [28], including post-operative surveillance following PCC/PGL resection [29] as well as for other tumors such as neuroblastoma [33,34].

The aim of this study was to assess the value of ^11^C-HED-PET/CT to diagnose or rule out PCC/PGL in complex clinical scenarios by allowing for tumor detection and/or characterization in patients with equivocal symptoms and/or biochemical and/or CT/MRI findings, in whom conventional work-up had failed to guide the clinical decision. 

## 2. Results

### 2.1. Baseline Patient Characteristics

Median age in the 102 patients was 58 ± 2 years, with a male to female ratio of 1:1. Results from genetic testing was available in only eight patients; Multiple Endocrine Neoplasia type 2A was diagnosed in three patients (patients 10, 11, 12, Table 3), SDHB-related PGL in three patients (patients 21, 23, 25, Table 4), one had Neurofibromatosis type 1 and one had Multiple Endocrine Neoplasia type 2B (patients 7 and 9, respectively, Table 3). The patients presented with symptoms suspicious for PCC/PGL (*n* = 68), elevated biochemistry (*n* = 29) or borderline biochemistry (*n* = 57) and radiologically uncharacterized tumors (*n* = 26, out of which 16 presented as incidentalomas), or a combination thereof. The tumors in the latter 26 patients had not been possible to characterize on CT/MRI based on general radiological appearance in combination with attenuation measurements, contrast medium washout and results of in- and out-of-phase MRI. Also, several patients harbored bilateral tumors. Because the previous conventional work-up for adrenal disease had failed to provide the diagnosis, or to rule out PCC/PGL, they subsequently underwent ^11^C-HED-PET/CT.

### 2.2. Diagnostic Performance

**Figure 1:**
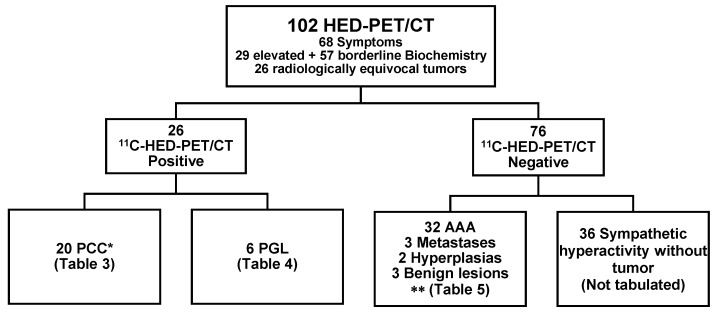
Flowchart of the 102 study patients. ^11^C-HED-PET/CT visualized 20 PCC (Table 3) and six PGL (Table 4) and ruled out PCC/PGL in 76 patients, including 40 patients with adrenal tumors (Table S1) and 36 without tumors. * including one false negative ^11^C-HED-PET/CT result, ** including one false positive PET/CT result. AAA; adrenocortical adenoma.

A flow chart of the study is presented in Figure 1. The results of ^11^C-HED-PET/CT are shown in Table 1 and Table 2. With correlation to a combined gold standard, comprising histopathology, biochemical diagnosis, findings at surgery and on radiological and clinical follow-up, ^11^C-HED-PET/CT in the 102 patients showed 96% sensitivity, 99% specificity, 96% positive predictive value and 99% negative predictive value (Table 1). When the ^11^C-HED-PET/CT results were strictly correlated to tumor histopathology, as the gold standard (*n* = 41), the sensitivity, specificity, positive predictive value and negative predictive value were 96%, 93%, 96% and 93%, respectively (Table 2).

### 2.3. Tabulated Results

Table 3 shows the results for the patients with histopathologically confirmed PCC (*n* = 20). Table 4 displays the results for the patients with extra-adrenal tumors, which on subsequent histopathology were diagnosed as PGLs (*n* = 6). Table S1 (Supplementary Materials) gives the results for the 40 patients with ^11^C-HED-negative adrenal tumors. The remaining 36/102 patients without an adrenal tumor on CT, and in whom ^11^C-HED-PET/CT ruled out PCC/PGL, are not tabulated.

Out of the 20 PCC patients (Table 3), eight had symptoms indicating catecholamine excess and 12 presented with adrenal incidentaloma on CT/MRI. The PCC was bilateral in four patients and unilateral in 16, of whom one patient had a PCC (^11^C-HED-positive) together with an adrenocortical adenoma (^11^C-HED-negative) in the same adrenal (collision tumor). The only patient for whom ^11^C-HED-PET/CT was false negative harbored a PCC in one adrenal gland and a hyperplasia in the other (Patient 19, Table 3). The PCCs measured 1–8 cm in size, with a mean of 2.9 cm.

Out of the six patients with histopathologically confirmed PGL (Table 4), four presented with symptoms and/or biochemistry indicating catecholamine excess and two with adrenal incidentaloma on CT/MRI. One patient (Patient 26, Table 4) with a ^11^C-HED-negative tumor in the neck was diagnosed with a ^11^C-HED positive metastasis to the right femur. The PGLs measured 4–7 cm in size, with a mean of 4.8 cm. 

Out of 40 patients with ^11^C-HED-PET/CT-negative adrenal tumors (Table S1, Supplementary Materials), nine patients with symptoms of catecholamines excess and equivocal biochemistry underwent surgery and the histopathological examination showed adrenocortical adenomas. CT-guided biopsy in two patients revealed in both metastasis from lung adenocarcinoma. Bilateral ^11^C-HED-positive adrenals were found in one patient who underwent surgery and multinodular hyperplasia was diagnosed an histopathology and constituted the only false positive ^11^C-HED-PET/CT result (Patient 45, Table S1). The diagnoses in the remaining 28 patients were based on extended radiological and clinical follow-up.

### 2.4. ^11^C-HED Accumulation and Uptake Measurements

**Figure 2:**
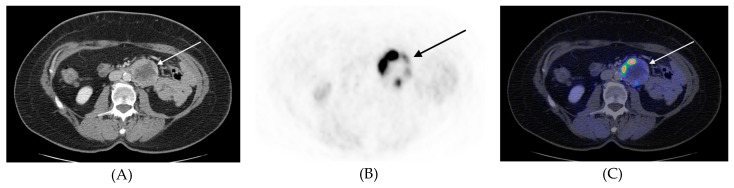
Transverse ^11^C-HED-PET/CT images, (**A**) CT, (**B**) PET, (**C**) PET/CT fusion, of patient #21 (Table 4) with a retroperitoneal paraganglioma left of the descending aorta (arrows). In this paraganglioma there was extended necrosis and merely peripheral tracer accumulation in the tumor.

^11^C-HED was found to generally accumulate throughout the extent of the whole tumor, but in five patients showed heterogeneous, predominately peripheral ^11^C-HED uptake, because of extended tumor necrosis (Figure 2). 

The ^11^C-HED*-*PET measurements are shown in Tables S2 and S3 (Supplementary Materials). For the PCCs and PGLs (*n* = 26), no correlation was found between tumor SUVmax and tumor size (*R^2^* = 0.05, *p* = 0.286) The SUVmax and the tumor-to-liver ratio was significantly higher in tumors larger than 4 cm (*p* = 0.045, *p* = 0.033).

The systolic BP was less than 140 mmHg in 34 patients, between 140 and 180 mmHg in 39 subjects and exceeded 180 mmHg in 24 patients. The tumor SUVmax and the tumor-to-liver ratio were unrelated to the systolic BP, and also to symptoms of catecholamine excess.

In PCC & PGL patients with ^11^C-HED-positive tumors, norepinephrine somewhat correlated with the systolic BP (*R^2^* = 0.22, *p* = 0.016) and increased in parallel. Patients with high norepinephrine had higher systolic BP (cutoff = twice the upper reference value, *p* = 0.025, cutoff = 10 times the upper reference value (*p* = 0.013). The tumor SUVmax and the tumor-to liver ratio was higher in patients with high norepinephrine (cutoff = 10 times the upper reference value, *p* = 0.009).

## 3. Discussion

In this study, ^11^C-HED-PET/CT in a cohort of 102 patients with suspected PCC/PGL, based on equivocal clinical symptoms and/or biochemical and/or radiological findings, showed 96% sensitivity, 99% specificity, 96% positive predictive value and 99% negative predictive value. These data provide additional evidence that ^11^C-HED-PET/CT can be used in the diagnostic work-up of patients with suspected PCC/PGL in whom the conventional clinical, biochemical and radiological work-up failed to provide diagnosis, or to exclude PCC/PGL.

Since 2005, our center has used ^11^C-HED-PET/CT as a problem-solving tool in patients with suspected PCC/PGL, for postoperative surveillance, therapy monitoring and diagnosis of recurrent disease. A combined evaluation of ^11^C-HED-PET and ^11^C-HED-PET/CT, performed for several of these indications, has shown favorable diagnostic capacity [26]. In the present work, we instead concentrated on exclusively evaluating ^11^C-HED-PET/CT performed for the sole purpose of primary diagnosis (or ruling out disease) in patients with suspected PCC/PGL. 

In this retrospective setting, ^11^C-HED was not compared with other PET tracers. ^11^C-HED is not generally available and the 20 min half-life of ^11^C is another inconveniency. PET tracers labeled with ^18^F or ^68^Ga (110 and 68 min half-life, respectively) are advantageous in this respect. The common metabolic PET tracer ^18^F-FDG shows low specificity and lower diagnostic yield than ^18^F-DOPA and ^68^Ga-DOTA-somatostatin analogs [35].^18^F-FDG has, however, been shown to be highly sensitive in the detection of *SDHB*-related PCCs/PGLs [36]. The sensitivity of ^18^F-DOPA-PET/CT depends on the tumor type and, in a direct comparison, ^68^Ga-DOTATATE has the advantage of fairly high availability and was better than ^18^F-DOPA at visualizing PCCs but less sensitive for HNPGLs [15]. In 101 PCC/PGL patients, ^18^F-DOPA showed 93% sensitivity and 88% specificity [37]. In a comparative PET/CT study in PCC/PGL patients, ^18^F-DA was considered the preferred tracer, followed by ^18^F-DOPA and ^18^F-FDG [22].

**Figure 3:**
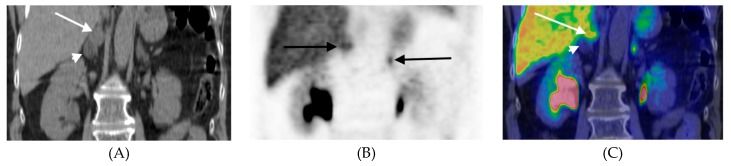
^11^C-HED-PET/CT, coronal mages, of patient #2 (Table 3) with a collision tumor in the right adrenal showing a cranial component with high tracer uptake comprising a PCC (arrows) and a caudal tumor portion representing an adrenocortical adenoma (arrow head). The normal contralateral adrenal is indicated with an arrow in the PET image. (**A**) CT, (**B**) PET, (**C**) PET/CT fusion.

CT/MRI characterization of PCC is a challenge because these tumors display a wide range of appearances [38]. PCC is therefore, in this sense, sometimes referred to as a chameleon among adrenal tumors. This was illustrated by the fact that the most abundant CT/MRI findings in our patients instead were tumor heterogeneity, necrosis and irregular margins. Interestingly, the CT/MRI findings were consistent with adrenocortical adenomas in nine patients (Table 2 and Table 4). ^11^C-HED-PET/CT was nevertheless performed because of equivocal biochemistry/symptoms to rule out PCC/PGL in other locations. An important example in this respect, is the ^11^C-HED-positive patient (2, Table 2) harboring a PCC together with a benign adrenocortical adenoma (“collision tumor”) (Figure 3).

Patients with biochemistry and/or symptoms consistent with PCC/PGL and the CT/MRI findings of an adrenal tumor usually undergo surgical resection. However, 16 such patients nevertheless underwent ^11^C-HED-PET/CT, because of bilateral tumors, to localize possible extra-adrenal lesions and to assess the local tumor extent. When a tumor cannot be localized by CT/MRI, despite biochemistry and/or symptoms consistent with PCC/PGL, ^11^C-HED-PET/CT can be useful to identify the PCC in cases of bilateral adrenal tumors and tumors of extra-adrenal origin. Further, ^11^C-HED-PET/CT was found instrumental for ruling out PCC/PGL in 78 of our patients, in whom CT could not provide firm evidence on the origin of the tumor. Only PCC was missed (patient 19, Table 3), and the only false positive PET/CT result was represented by a nodular hyperplasia (patient 45, Table S1).

**Figure 4:**
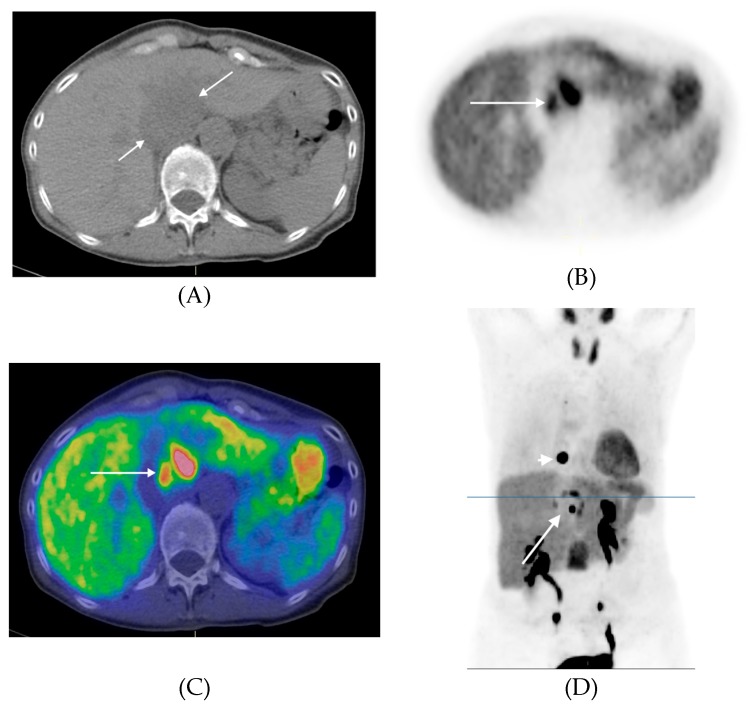
^11^C-HED-PET/CT of patient #24 (Table 4) with a retroperitoneal paraganglioma in front of the descending aorta with high heterogenous tracer uptake (long arrows) and a metastasis in the transverse process of the thoracic vertebra 10 shown in D (short arrow). In the coronal PET image an additional vertebral metastasis in the first lumbar vertebra is seen projected between the kidneys. (**A**) Transversal CT, (**B**) Transversal PET, (**C**) Transversal PET/CT fusion, (**D**) Coronal PET (Maximum Intensity Projection). The level of the transversal images are indicated in the coronal PET image (line).

Interesting findings were encountered in a patient (26, Table 4) with a ^11^C-HED negative primary HNPGL but with a ^11^C-HED-positive metastasis in the left femur. Histopathology showed a parasympathetic paraganglioma with high proliferation (Ki-67 index 20%). Parasympathetic paraganglioma cannot be expected to be ^11^C-HED-positive and the fact that the metastasis showed ^11^C-HED uptake is intriguing and difficult to explain. Notably, ^18^F-FDG-PET/CT in this patient showed both lesions to be ^18^F-FDG avid. Sympathetic paragangliomas were, however, all visualized ^11^C-HED, including metastases (Figure 4).

Some weaknesses in our study were its retrospective and single design and the fact that surgical and histopathological confirmation of the ^11^C-HED-PET/CT findings was not provided for all patients. However, we believe that the extended clinical, biochemical and imaging follow-up of the patients provided us with unique material that may compensate for this absence of histopathological confirmation. We provide data from morphological imaging (CT) but no comparison with molecular imaging, and a prospective comparative PET/CT study primarily with ^18^F-FDG and ^68^Ga-DOTA-somatostatin analog is therefore warranted.

## 4. Patients and Methods

### 4.1. Patients

This was a retrospective cohort study of 102 patients investigated at the Department of Nuclear Medicine, Uppsala University Hospital, Uppsala, Sweden. The study was approved by the Regional Ethics Committee (No. 2012/422). All patients that underwent ^11^C-HED-PET/CT were screened for inclusion using information available through the digital radiological information and picture archive and retrieval systems (RIS-PACS). We selected those who underwent PET/CT examination between March 2005 to September 2017. Patients having undergone PET/CT for postoperative surveillance were excluded. Clinical, biochemical and radiological imaging follow-up data were retrieved from the RIS-PACS and from the hospital’s digital patient record system. 

### 4.2. ^11^C-Hydroxy-ephedrine-PET/CT Examination

From March 2005, a GE Discovery ST PET/CT scanner was used (General Electric Medical Systems, Milwaukee, WI, USA). The PET scanner produced 47 slices with a 157 mm axial field of view (FOV) and 700 mm trans-axial FOV. Patients were injected with approximately 800 MBq of ^11^C-HED and static whole-body images were obtained 20 min later, from the base of the skull to the upper thighs. The spatial resolution was equal to that of the individual crystal size in the block, approximately 5‒6 mm. A non-contrast-enhanced, low-radiation-dose CT examination was performed before the PET acquisition for attenuation correction of the PET images and for anatomical correlation of the PET findings.

### 4.3. Image Analysis and Interpretation

Qualitative image interpretation and PET measurements were first performed by one of the authors (ARV) and then again in a second reading session together with a radiologist with 25 years of PET experience (AS) and a common consensus was reached regarding the image findings. Image reading and PET measurements were performed on a computer workstation connected to the hospital’s PACS system. Any focal accumulation of ^11^C-HED exceeding the normal physiological uptake was regarded as pathological. A tumor with a ^11^C-HED uptake higher than that of the contralateral normal adrenal was considered ^11^C-HED-positive and consistent with PCC/PGL. Moderate physiological tracer uptake in regions such as the salivary glands, myocardium, liver, spleen, pancreas and normal adrenal medulla was disregarded.

Quantification of ^11^C-HED uptake in tumors utilized the standardized uptake value (SUV), which was calculated for each pixel by dividing its radioactivity concentration (Bq/mL) by the injected radioactivity (Bq) per gram of body-weight. Regions of interest (ROIs) were drawn manually to measure SUVmax (the maximum pixels in the ROI) in each lesion. Also, as a normal tissue reference, a 2-cm circular ROI was drawn in the posterior part of the right liver lobe and the SUVmean was registered. The tumor-to-liver ratio was calculated as tumor SUV_max_/normal liver SUV_mean_. In patients with adrenal tumors, the SUV_max_ of the contralateral normal adrenal was additionally assessed and the tumor-to normal-adrenal ratio was calculated.

Plasma and urinary epinephrine and norepinephrine samples were for matters of comparison normalized to the upper reference value and correlated to^11^C-HED uptake measurements.

### 4.4. Statistical Analysis

Data were presented as mean ± standard deviation (SD). Differences in means between groups were evaluated using a *t*-test assuming unequal variances. Pearson’s correlation test was performed to evaluate the relationship between variables. All statistical analyses were performed in IBM^®^ SPSS^®^ Statistics V.24 and JMP 13.1 (SAS Institute, Inc., Cary, NC, USA). *p* < 0.05 was regarded significant.

## 5. Conclusions

In conclusion, ^11^C-HED-PET/CT is a valuable tool in complex clinical scenarios, with findings of biochemistry/symptoms/radiology suspicious of PCC/PGL, when conventional work-up fails to diagnose or rule out disease. ^11^C-HED uptake measurements were, however, unable to assist with the tumor characterization.

## Figures and Tables

**Table 1:** ^11^C-HED-PET/CT results in all 102 patients with correlation to findings at surgery, histopathology, biochemistry, clinical and radiological follow-up (combined gold standard).

		Gold standard	
		Positive	Negative
^11^C-HED-PET/CT	Positive	25	1
	Negative	1	75

**Table 2:** ^11^C-HED-PET/CT results in the 41 patients operated on, with correlation to findings at surgery and histopathology (gold standard).

		Gold standard	
		Positive	Negative
^11^C-HED-PET/CT	Positive	25	1
	Negative	1	14

**Table 3:** CT characteristics and ^11^C-HED-PET/CT parameters in the 20 patients with histopathologically confirmed pheochromocytoma (PCC) including 19 who were correctly characterized by ^11^C-HED-PET/CT and one false negative ^11^C-HED-PET/CT result (*). HT; hypertension, NET; neuroendocrine tumor, BP; blood pressure, CECT; contrast-enhanced CT, AAA; adrenocortical adenoma, L; left, R; right, A; epinephrine, NA; nor-epinephrine, A-Ref; ratio of value and upper normal reference range value, NA-Ref; ratio of value and upper normal reference range value.

Pat No.	Age	Sex	Clinical Information	Incidenta-loma	Systolic BP	Diagnosis (PAD)	NA-Ref	A- Ref	A/N Ratio
1	45	F	Sweating, palpitations anxiety	Y	220	L PCC R AAA	11.5	1.00	0.09
2	73	F	Palpitations, headache, HT, alpha blocker	Y	150	R PCC+AAA	2.00	3.50	1.75
3	67	F	Sweating, headache, alpha blocker	Y	150	R PCC L AAA	1.33	1.00	0.75
4	48	F	Anxiety, palpitations, muscle fasciculations, alpha blocker	Y	150	PCC	12.8	1.00	0.08
5	85	F	Rectal cancer Incidentaloma	Y	140	PCC with cystic areas	10.7	1.00	0.09
6	53	F	Polycystic kidney disease, HT	Y	180	PCC	1.00	5.00	5.00
7	52	M	No symptom	Y	190	PCC	2.00	1.44	0.72
8	71	F	Breast cancer, small-intestinal NET	Y	150	PCC	0.56	0.51	0.92
9	19	F	Bilateral incidentalomas	Y	180	PCC	1.83	3.50	1.91
10	58	F	Palpitations, alpha blocker	Y	130	PCC	1.33	1.00	0.75
11	28	F	No symptoms	Y	120	PCC	1.56	2.13	1.37
12	30	F	Palpitations, panic attack	N	110	PCC	1.33	3.02	2.27
13	72	F	Sweating, alpha blocker	N	220	PCC	3.74	1.67	0.45
14	50	F	Palpitations, headache, HT	N	215	PCC	61.7	95.0	1.54
15	59	M	Incidentaloma	Y	200	PCC	1.00	2.50	2.50
16	42	F	Palpitations, sweating, headache, tremor	N	130	PCC with cystic areas	2.68	9.80	3.66
17	58	F	Sweating, palpitations HT, alpha blocker	N	170	PCC with necrosis	8.83	39.5	4.47
18	64	M	HT, alpha blocker	N	220	PCC with necrosis	22.3	107	4.77
19	61	F	Headache, flushing, sweating, palpitations, alpha blocker	N	230	L PCC* R hyperplasia	11.4	3.89	0.34
20	65	F	Sweating, palpitations, alpha blocker	N	140	PCC	2	1	0.5

**Table 4:** CT characteristics and PET/CT parameters in six patients with ^11^C-HED uptake in extra-adrenal sites that, after surgery, were histopathologically confirmed as paragangliomas (PGL). HT; hypertension, BP; blood pressure, ND; not done, HU; Hounsfield Units, CECT; contrast-enhanced CT, A; epinephrine, NA; norepinephrine.

Pat No.	Age	Sex	Clinical Information	Inci-denta-loma	Systolic BP	Location	Diagnosis (PAD)	P-met-tyramine (< 0.2)	NA	A	A/NARatio
21	60	F	Back pain	Y	145	Para-aortic	PGL	0.4	6.17	0.67	0.11
22	34	F	Headache, palpitations	N	220	Pre-aortic	PGL	ND	18.3	0.67	0.04
23	16	M	Palpitations, headache, HT	N	180	Pre-aortic	PGL	ND	1.67	2.00	1.20
24	71	M	Abdominal pain	N	130	Pre-aortic	PGL	1.1	0.50	0.67	1.33
25	56	M	Abdominal pain	Y	180	Pre-aortic	PGL	0.5	2.50	0.67	0.27
26	70	M	Unclear symptoms	N	135	Neck	PGL	0.8	2.33	0.67	0.29

## Supplementary Materials

The following are available online at https://www.mdpi.com/2072-6694/11/6/847/s1, Table S1: CT and PET/CT parameters in 40 patients with 11C-HED-negative tumors, Table S2: Size and 11C-HED accumulation (standardized uptake value, SUV) in 11C-HED-PET/CT positive tumors, Table S3: Statistical analysis of various parameters using t-test assuming unequal variances.
