# Supplementary material for: 11C-hydroxy-ephedrine-PET/CT in the Diagnosis of Pheochromocytoma and Paraganglioma
Source: Cancers (Basel). 2019 Jun 19;11(6):847. doi: 10.3390/cancers11060847 (PMC6627429; doi:10.3390/cancers11060847)
Supplement: Supplementary file 1 [file cancers-11-00847-s001.pdf]

Article

^{11}C-hydroxy-ephedrine-PET/CT in the Diagnosis of Pheochromocytoma and Paraganglioma

Achyut Ram Vyakaranam [1,4,*], Joakim Crona [2,3], Olov Norlén [4], Per Hellman [4] and Anders Sundin [1,4]

[1] Section of Radiology & Molecular Imaging, Department of Surgical Sciences, Uppsala University, Akademiska Sjukhuset, SE-751 85 Uppsala, Sweden; anders.sundin@radiol.uu.se
[2] Department of Medical Sciences, Uppsala University, Akademiska Sjukhuset, SE-751 85 Uppsala, Sweden; joakim.crona@medsci.uu.se
[3] Department of Immunology, Genetics and Pathology; Uppsala University, Akademiska Sjukhuset, SE-751 85 Uppsala, Sweden
[4] Department of Surgical Sciences, Uppsala University, Akademiska Sjukhuset, SE-751 85 Uppsala, Sweden; olov.norlen@surgsci.uu.se (O.N.); per.hellman@surgsci.uu.se (P.H.)
* Correspondence: achyutram.vyakaranam@surgsci.uu.se

Received: 11 June 2019; Accepted: 16 June 2019; Published: 19 June 2019

Table S1. CT and PET/CT parameters in 40 patients with ^{11}C-HED-negative tumors. PCC;pheochromocytoma, PGL;paraganglioma, HT;hypertension, NET;neuroendocrine tumor, AAA;adrenocortical adenoma, ND;not done, BP;blood pressure, L;left, R;right, Retroper;retroperitoneum, HU;Hounsfield Units, CECT;contrast-enhanced CT, A;epinephrine, NA;norepinephrine, FU;follow-up.

Pat No.	Age	Sex	Clinical information	Inci-denta-loma	Indication for 11C-HED-PET/CT	Systolic BP	Location L/R	Size (L,R) cm		Attenuation (L, R) (HU)	Tumor SUVmax	Normal adrenal SUVmax	Normal liver SUVmean	Tumor/ Normal adrenal Ratio	Tumor/ Liver Ratio	Final Diagnosis (PAD/FU)	U-NA (< 400)	U-A (< 90)	P-Met-NA (< 0.6)	P-Met –A (< 0.3)	P-met-tyramine (< 0.2)
27	63	F	Palpitations, sweating, HT	N	Biochemistry	210	L	2.5		-10	3.5	3.5	4.6	1.00	0.76	AAA	635	92	0.6	0.2	3
28	54	M	Incidentaloma	Y	HT, biochemistry	170	L	3.5		-20	8.2	9.8	4.5	0.84	1.82	AAA	550	77	0.4	0.2	
29	65	M	No symptoms	Y	Rule out PCC	140	L	2.5		-20	12.6	10	4.6	1.26	2.74	Benign on FU (AAA)			0.8	0.2	
30	55	M	No symptoms	Y	Biochemistry	140	L/R	3.5	1	-20	6.5	4.8	4.4	1.35	1.48	Bilateral AAA			3	0.2	
31	61	F	Insulinoma Incidentaloma	Y	Rule out PCC	150	L	1.5		-12	10.4	8.1	4.3	1.28	2.42	Benign on FU (AAA)	980	130			
32	53	F	Incidentaloma, biochemistry	Y	Rule out MTC metastasis	160	L	1		>10	11.4	7.6	6.6	1.50	1.73	MTC metastasis (FU)			1	0.2	
33	68	M	Headache, HT	N	Rule out PCC	200	L	1		<0	6.5	5.5	4.4	1.18	1.48	Benign on FU (AAA)			1	0.2	
34	59	F	Incidentaloma	Y	Rule out PCC	160	R		1.5	>10	4.3	4	4.3	1.08	1.00	Benign on FU (AAA)			1.4	0.2	

35	58	F	Headache, abdominal pain	Y	HT, biochemistry	210	R		1.5	10	7.7	3.6	5	2.14	1.54	Cyst and AAA			0.5	0.3	2.6
36	47	F	Incidentaloma	Y	Rule out lung cancer metastasis	145	L	1.5		CECT	4	4.6	4	0.87	1.00	Lung cancer metastasis (Biopsy)			0.6	0.2	
37	58	F	Incidentaloma	Y	Rule out lung cancer metastasis	145	R		4.5	ND	ND	7.7	5	ND	ND	Lung cancer metastasis (Biopsy)			0.3	0.2	
38	60	F	Flushes, panic attack	N	Rule out PGL	230	Abdomen	4		ND	6	5	4.5	1.20	1.33	Benign on FU (Hematoma)	1212	59	0.6	0.2	
39	58	F	Palpitations, HT	Y	Rule out PCC	180	R		1.2	25	6.6	8	5	0.83	1.32	Benign on FU (AAA)	1430	96	0.2	0.2	
40	62	M	Panic attack, biochemistry	Y	Rule out PCC	210	L	1.5		>10	11.4	7.9	5	1.44	2.28	Benign on FU (hyperplasial)	1100	150	0.3	0.3	
41	66	M	No symptoms	Y	Rule out PGL	150	Retroper.	6		ND	4	3.2	4.8	1.25	0.83	Benign on FU (Neurofibroma)			0.4	0.3	
42	63	M	No symptoms	Y	Biochemistry	130	R		2.5	<10	3	4.3	6.2	0.70	0.48	AAA	560	95	1	0.2	1.5
43	66	M	Biochemistry	Y	Rule out Bilateral PCC	160	L/R	2	1.5	0 8	4.9	3	5.5	1.63	0.89	Benign on FU (AAA)	620	46	1	0.2	
44	72	M	Palpitations	Y	Rule out Bilateral PCC	160	L/R	3	1	CECT	9.9	9.3	5	1.06	1.98	AAA		180	0.3	0.2	
45	60	F	HT	Y	Rule out bilateral PCC	170	L/R	2	2	CECT	8.3	5.3	4	1.57	2.08	L+R nodular hyperplasia		195	0.3	0.2	
46	54	F	Incidentaloma, biochemistry	Y	Rule out bilateral PCC	140	L/R	2.5	1	-90	9	6	7.3	1.50	1.23	Benign on FU (Myelolipoma)			1.5	0.2	
47	59	F	Incidentaloma	Y	Rule out bilateral PCC	175	L/R	1.2	1	<0	4.8	4.2	4.5	1.14	1.07	Benign on FU (AAA)	580	100			
48	50	M	Incidentaloma	Y	Rule out bilateral PCC	170	L/R	2.5	1	2 28	5	6.5	4	0.77	1.25	Benign on FU (AAA)			0.4	0.2	
49	64	M	Incidentaloma	Y	Rule out bilateral PCC	No info	L/R	1	2	<0	11.4	10	5	1.14	2.28	Benign on FU (AAA)			0.4	0.2	
50	72	F	Incidentaloma	Y	Rule out bilateral PCC	no info	L/R	2.5	1	<0	9	8	5.4	1.13	1.67	Benign on FU (AAA)			0.3	0.2	
51	54	F	Incidentaloma	Y	HT and bilateral incidentaloma	160	L/R	2	2	<10	6.8	8.2	6.5	0.83	1.05	Bilateral AAA			0.3	0.2	
52	64	M	Unclear symptoms	N	Rule out bilateral PCC	no info	L/R	3	1	-20	9	15.5	5.4	0.58	1.67	Benign on FU (AAA)			0.3	0.2	
53	70	M	Unclear symptoms	N	Biochemistry	180	L	1		>10	4.5	2.8	4	1.61	1.13	Benign on FU (AAA)			1.1	0.2	
54	73	F	Headache, HT	N	HT, Biochemistry	190	L	2.5		-20	7.5	8.5	6.5	0.88	1.15	AAA			1.4	0.2	
55	59	M	Tachycardia, headache, HT	N	Biochemistry	175	L	3		CECT	6.9	6.8	5	1.01	1.38	AAA			0.3	0.2	1.5
56	77	M	Sweating, HT	N	Biochemistry	180	L	1		<0	5	5	5.8	1.00	0.86	Benign on FU (AAA)			1	0.2	
57	55	M	Polydipsia, HT	N	Rule out PCC	230	L	2		-10	4.2	6	4	0.70	1.05	Benign on FU (AAA)	625	87	0.3	0.2	
58	51	M	Unclear symptoms	N	Biochemistry	160	L	1		>10	8.6	8.7	4.5	0.99	1.91	Benign on FU (AAA)	670	120	0.3	0.2	
59	41	M	HT	N	HT, biochemistry	190	L	3		26	5.6	9.9	5	0.57	1.12	AAA	1150	60	0.7	0.2	

60	63	F	Incidenataloma	Y	Rule out lung cancer metastasis	140	L	1		CECT	12.6	11	6.8	1.15	1.85	Benign on FU (AAA)			0.5	0.2	
61	56	F	Incidenataloma	Y	Rule out PCC	150	L	1.5		ND	15	16.5	8	0.91	1.88	Benign on FU (AAA)			0.3	0.2	
62	66	M	Incidenataloma	Y	Rule out lung cancer metastasis	180	R		3	-10	5.6	5.5	5.5	1.02	1.02	AAA			0.3	0.2	
63	54	M	Sweating, flushes, palpitations	N	Biochemistry	130	R		2	-10	5.6	5.4	5	1.04	1.12	Benign on FU (AAA)	1560	145	2.2	0.8	
64	48	M	Sweating	N	Biochemistry	140	R		1	30	3.2	3.6	2.9	0.89	1.10	AAA	800	66			2.7
65	61	F	HT, arrythmia	N	Rule out PCC	175	L	2		CECT	10.1	7.2	5	1.40	2.02	Benign on FU (AAA)			0.6	0.2	
66	57	F	HT, palpitations sweating	N	Biochemistry	160	R	1	1	ND	9.7	10.7	8.23	0.91	1.18	Benign on FU (AAA)			1.9	0.2	

cancers MDPI

Table S2. Size and ^{11}C-HED accumulation (standardized uptake value, SUV) in ^{11}C-HED-PET/CT positive tumors.

	Tumor size (cm)	Tumor SUVmax	Normal adrenal SUVmax	Normal Liver SUVmean	Tumor-to-normal adrenal-ratio	Tumor-to-normal liver-ratio
Mean±SD	3.35±1.95	14.83±8.78	8.36±3.31	5.05±0.89	2.05±1.64	3.03±1.89
Min	1.00	5.20	3.80	3.40	0.67	1.00
Max	8.00	46.50	17.00	7.90	7.05	8.94

Table S3. Statistical analysis of various parameters using t-test assuming unequal variances.

	Tumor SUV_{max}	Systolic BP	T/L
Patients with sympathetic hyperactivity versus those without sympathetic hyperactivity	0.21	0.9	0.15
Tumor size versus SUV_{max}, systolic BP and T/L ratio			
Cut-off = 2 cm	0.07	0.23	0.03
Cut-off = 4 cm	0.045*		0.033*
Low NA vs High NA			
Cut-off = > 2 times reference	0.12	0.024*	0.044*
Cut-off = > 10 times reference	0.035*	0.015*	0.009*
High systolic BP			
Cut-off = >180	0.36		0.26
Cut-off = >200	0.37		0.20

* $p < 0,05$, BP;blood pressure, NA;norepinephrine, SUVmax;maximum standard uptake value, T/L;tumor-to-liver ratio (ratio of tumor SUVmax and liver SUVmean).

Table 3. CT characteristics and PET/CT parameters in the 20 patients with ^{11}C-HED- positive adrenal tumors which after surgery were histopathologically confirmed as pheochromocytoma (PCC). HT;hypertension, NET;neuroendocrine tumor, BP;blood pressure, CECT;contrast-enhanced CT. AAA;adrenocortical adenoma, ND;not done, L;left, R;right, A;epinephrine, NA;nor-epinephrine, *;Lesion represents an AAA, A-Ref;value/upper normal reference value; NA-Ref;value/upper normal reference value.

Pat No.	Age	Sex	Clinical information	Incidentaloma	Systolic BP	Location	Size (L, R) cm		Attenuation (L,R) (HU)		Tumor SUVmax	Normal adrenal SUV_{max}	Normal liver SUV_{mean}	Tumor/Adrenal Ratio	Tumor/Liver Ratio	Diagnosis (PAD)	U-NA (<400)	U-A (<90)	U-Met-NA (<2.5)	U-Met-A (<1.5)	P-Met-NA (<0.6)	P-Met–A (<0.3)	NA-Ref	A-Ref	A(NRatio
1	45	F	Sweating, palpitations anxiety	Y	220	L	6	1*	10	-20	23	3.8	3.4	6.05	6.76	L PCC R AAA	12000	130			6.9	0.2	11.5	1.00	0.09
2	73	F	Palpitations, headache, HT, alpha blocker	Y	150	R		1.51*		10-15	7.7	7.2	6	1.07	1.28	R PCC+AAA					1.2	0.7	2.00	3.50	1.75
3	67	F	Sweating, headache, alpha blocker	Y	150	R	1*	2	CECT		12	9.8	5	1.22	2.40	R PCC L AAA					0.8	0.2	1.33	1.00	0.75
4	48	F	Anxiety, palpitations, muscle fasciculations, alpha blocker	Y	150	L	4		CECT		21.8	5.7	4.2	3.82	5.19	PCC	10627				7.7	0.2	12.8	1.00	0.08
5	85	F	Rectal cancer Incidentaloma	Y	140	L	5		ND		21.1	6.3	4.7	3.35	4.49	PCC with cystic areas	680				6.4	0.2	10.7	1.00	0.09
6	53	F	Polycystic kidney disease, HT	Y	180	L	4.5		40		19.8	8.2	7	2.41	2.83	PCC					0.6	1	1.00	5.00	5.00

7	52	M	No symptom	Y	190	R		2	>20		25	5	4.9	5.00	5.10	PCC	800	130					2.00	1.44	0.72
8	71	F	Breast cancer, small-intestinal NET	Y	150	L	1		>20		21.9	7.3	7.9	3.00	2.77	PCC	222	46	1.5	0.8			0.56	0.51	0.92
9	19	F	Bilateral incidentalomas	Y	180	L/R	2	2	>20		21	17	5	1.24	4.20	PCC					1.1	0.7	1.83	3.50	1.91
10	58	F	Palpitations, alpha blocker	Y	130	L/R	2	2	ND		13.2	12.8	4.3	1.03	3.07	PCC					0.8	0.2	1.33	1.00	0.75
11	28	F	No symptoms	Y	120	L/R	2	2.5	>20		6.5	6.5	4.6	1.00	1.41	PCC			3.9	3.2			1.56	2.13	1.37
12	30	F	Palpitations, panic attack	N	110	L/R	2	2	30	30	13.3	8.3	5.3	1.60	2.51	PCC	354	272			0.8		1.33	3.02	2.27
13	72	F	Sweating ,alpha blocker	N	220	R		2.5	ND		12.6	8.3	5.7	1.52	2.21	PCC	1495	150					3.74	1.67	0.45
14	50	F	Palpitations, headache, HT	N	215	R		6		>20	12.9	9.5	4	1.36	3.23	PCC					37	19	61.7	95.0	1.54
15	59	M	Incidentaloma	Y	200	L	2		>20		5.2	3.8	4.4	1.37	1.18	Benign PCC	652	160			0.6	0.5	1.00	2.50	2.50
16	42	F	Palpitations, sweating, headache, tremor	N	130	L	6		CECT		8.2	12.3	6	0.67	1.37	PCC with cystic areas			6.7	14.7			2.68	9.80	3.66
17	58	F	Sweating, palpitations HT, alpha blocker	N	170	L	8		ND		13.7	8.2	5.5	1.67	2.49	PCC with necrosis			13.1	29.7	5.3	7.9	8.83	39.5	4.47
18	64	M	HT, alpha blocker	N	220	R		6	ND		31	6.5	4.5	4.77	6.89	PCC with necrosis					13.4	21.3	22.3	107	4.77

19	61	F	Headache, flushing, sweating, palpitations, alpha blocker	N	230	L	3	1	38		5.2	17.7	5	5.36	3.54	L PCC R hyperplasia	4567	350					11.4	3.89	0.34
20	65	F	Sweating, palpitations, alpha blocker	N	140	R		2		24	8.4	4.3	4	1.95	2.1	PCC					1.2	0.3	2	1	0.5

Table 4. CT characteristics and PET/CT parameters in 6 patients with ^{11}C-HED uptake in extra-adrenal sites which after surgery were histopathologically confirmed as paragangliomas (PGL). HT;hypertension, , BP;blood pressure, ND;not done, HU;Hounsfield Units, CECT;contrast-enhanced CT, A;epinephrine, NA;norepinephrine.

Pat No.	Age	Sex	Clinical information	Inci-denta-loma	Systolic BP	Location	Tumor size (cm)	Attenuation (HU)	Tumor SUVmax	Normal adrenal SUVmax	Normal liver SUVmean	Tumor/ Normal adrenal Ratio	Tumor SUVmax/ Liver SUVmean Ratio	Diagnosis (PAD)	U-NA (< 400)	U-A (< 90)	P-Met-NA (< 0.6)	P-Met-A (< 0.3)	P-met-tyramine (< 0.2)	NA	A	A/NA Ratio
21	60	F	Back pain	Y	145	Para-aortic	5	CECT	46.5	6.6	5.2	7.05	8.94	PGL			3.7	0.2	0.4	6.17	0.67	0.11
22	34	F	Headache, palpitations	N	220	Pre-aortic	4	>30	14.5	11.4	5	1.27	2.90	PGL	6223	90	11	0.2		18.3	0.67	0.04
23	16	M	Palpitations, headache, HT	N	180	Pre-aortic	4	>30	6.2	6.3	4.4	0.98	1.41	PGL			1	0.6		1.67	2.00	1.20
24	71	M	Abdominal pain	N	130	Pre-aortic	7	ND	12.2	7.7	4.2	1.58	2.90	Metastatic PGL			0.3	0.2	1.1	0.50	0.67	1.33
25	56	M	Abdominal pain	Y	180	Pre-aortic	4	>50	8.8	6.1	5.4	1.44	1.63	Metastatic PGL	595	37	1.5	0.2	0.5	2.50	0.67	0.27
26	70	M	Unclear symptoms	N	135	Left femur	4.5	ND	4.6	4	6	1.50	1.00	Metastatic PGL			1.4	0.2	0.8	2.33	0.67	0.29

cancers

MDPI

© 2019 by the authors. Submitted for possible open access publication under the terms and conditions of the Creative Commons Attribution (CC BY) license (http://creativecommons.org/licenses/by/4.0/).
